# Survivorship care guidance in a diverse cancer survivor population: Cancer Registry for Understanding and Improving Survivorship Experiences (CRUISE) Study

**DOI:** 10.1007/s11764-026-01970-1

**Published:** 2026-01-28

**Authors:** Scarlett Lin Gomez, Salma Shariff-Marco, Alyssa Cortella, Debora Oh, Pari Srivastava, Vlad Honcharov, Christine Duffy, Zinnia Loya, Michelle Wadhwa, Kathie Lau, Kristan Olazo, Niharika Dixit, Urmimala Sarkar

**Affiliations:** 1 Helen Diller Family Comprehensive Cancer Center, University of California, San Francisco, USA; 2 Department of Epidemiology & Biostatistics, University of California, 550 16th St. 2nd Floor, San Francisco, CA 94158 USA; 3 University of California, San Diego, USA; 4 Division of General Internal Medicine, University of California, San Francisco, USA; 5 Zuckerberg San Francisco General Hospital,, San Francisco, USA; 6 Division of Hematology/Oncology, University of California, San Francisco, USA

**Keywords:** Survivorship care plans, Race and ethnicity, Socioeconomic status, Disparities

## Abstract

**Purpose:**

Using a population-based approach, the CRUISE Study is designed to understand disparities in receipt of survivorship care guidance among medically under-resourced cancer survivors.

**Methods:**

Eligible participants identified from the Greater Bay Area Cancer Registry were those newly diagnosed with first primary stage I–III breast or colorectal cancer. Patients from medically under-resourced communities, defined on the basis of ethnic minority group, underinsurance, and/or living in a low socioeconomic status (SES) neighborhood, were oversampled. Using data derived from patient surveys, cancer registry, and geospatial linkage, patient, facility, and neighborhood attributes were assessed for associations with receipt of survivorship care guidance using univariate and multivariable log binomial regression models.

**Results:**

Of 867 participants (332 breast cancer, 474 colorectal cancer), 62% identified with a non-White racial/ethnic group, 20.6% resided in a low SES neighborhood, and 9.3% were uninsured or publicly insured. Overall, 72.2% received survivorship care guidance. In a multivariable model, female breast cancer survivors relative to male colorectal cancer survivors (RR: 0.89, 95% CI: 0.81–0.97), and unemployed (RR: 0.80, 95% CI: 0.68–0.94) and retired (RR: 0.85, 95% CI: 0.77–0.95) versus employed people remained significantly less likely to receive guidance. Those who strongly agreed as well as those who disagreed that they could get medical care without financial setbacks were also significantly more likely to have received guidance than those who neither agreed nor disagreed (RR: 1.28, 95% CI: 1.09–1.51 for strongly agreed; RR: 1.22, 95% CI: 1.01–1.46 for disagreed).

**Conclusions:**

The CRUISE Study demonstrated feasibility in accruing a population-based sample of cancer survivors with over-representation of medically under-resourced patients.

**Implications for Cancer Survivors:**

A high proportion received survivorship guidance, including survivorship care plans or post-treatment guidance, but some variations were found across some patient characteristics.

**Supplementary Information:**

The online version contains supplementary material available at https://doi.org/10.1007/s11764-026-01970-1.

## Introduction

There are an estimated 18.1 million cancer survivors in the United States (US), projected to increase to 26 million by 2040 [1]. Breast and colorectal cancer survivors account for approximately 30% of this population [2]. Early cancer detection and advances in treatment for both breast and colorectal cancers have resulted in improved survival rates and increased lifespan for cancer survivors [3]. Thus, there is an increasing and timely need for research and clinical care paradigms that adequately address the unique needs of this growing population. The comprehensive care of cancer survivors must include monitoring for recurrence or cancer-related complications, managing late and long-term treatment-related symptoms, improving health-related quality of life (HRQOL), regular primary or preventative care, and addressing the psychosocial burdens associated with cancer diagnosis, treatment across the survivorship period [4].

These cancer survivorship outcomes vary across sociodemographic groups, including those defined by race, ethnicity, income, and/or English proficiency, with cancer survivors who are considered economically or socially marginalized and medically under-resourced experiencing disparities across domains of survivorship care [5]. These include disparities in various domains of HRQOL, including physical, social, and mental functioning [6]; intensity of cancer surveillance [7]; and management of symptoms and comorbidities [8]. For example, Black cancer survivors diagnosed with breast cancer are more likely to experience poor physical function [9] and Latina breast cancer survivors are less likely to have access to post treatment surveillance and more likely to experience psychological distress [10].

Patients and families need guidance for addressing future survivorship care needs when they complete active treatment. To meet this need, the 2006 Institute of Medicine (IOM) report “From Cancer Patient to Cancer Survivors: Lost in Transition” recommended that patients receive a survivorship care plan (SCP), a comprehensive care summary and follow-up plan, to improve follow-up cancer care as well as long-term care and HRQOL. Per the IOM, SCPs must contain information about the patient’s cancer type and treatment with associated side effects, recommended follow-up timing for care, recommendations for preventative care, and psychosocial services [11]. From 2012–2019, the Commission on Cancer (COC) included a standard that all eligible cancer survivors should receive an SCP [12]. SCPs were intended to be reviewed by the patient and oncology treatment team at the conclusion of their active cancer treatment while they could be on maintenance therapy or endocrine therapy for breast cancer [13]. Prior studies demonstrated that SCP content and delivery vary across institutions [14] and sociodemographic groups. A recent scoping review demonstrated lower receipt of SCPs among patients of lower income, from ethnic minoritized groups, and/or rural areas, but that patients from these groups continue to have unmet needs in their survivorship care. This review suggests that while SCPs are no longer a required standard of care, they may thus still be beneficial for underserved patient populations [15]. Researchers exploring the survivorship needs of Black breast cancer survivors found that existing SCP templates lack adequate content on health history, comorbidities, and culturally responsive information, such as referrals for providers who are culturally competent and knowledgeable about issues that might be more common among Black populations [16]. Furthermore, research on the information needs of Chinese American breast cancer patients showed that in order to be useful and relevant, SCPs must include culturally relevant information, such as traditional food remedies and Chinese herbal medicine [17].

Across a range of studies, SCPs did not improve patient-reported outcomes including patient satisfaction with follow-up care, survivorship care or cancer-related knowledge, self-efficacy, cancer-related distress, anxiety, or depression [12]. Thus, the requirement for SCPs was dropped from COC accreditation in 2019. However, cancer survivors and family members continue to report informational needs about their future care emphasizing the need for SCPs [18]. While the majority of oncologists reported in a survey that they discussed survivorship care guidance, only very few reported delivering SCPs [19]. This suggests SCPs alone do not fully capture post-treatment care.

A goal of the CRUISE study was to take a comprehensive, multi-level approach to examine the implementation of, and a range of patient experiences with, SCPs and survivorship care guidance. We conducted a population-based study of breast and colorectal cancer survivors in the Greater San Francisco Bay Area, with overrepresentation of medically under-resourced populations including minoritized racial/ethnic groups, underinsured patients, and those residing in low socioeconomic neighborhoods. We assessed their receipt and use of SCPs or survivorship care guidance and impact of SCPs/care guidance on key patient-reported outcomes. Here, we describe our population-based approach and key results from the patient survey regarding multilevel factors associated with receipt of survivorship care information.

## Methods

### Study sample

Participants were identified from the Greater Bay Area Cancer Registry (GBACR), which includes nine counties in northern California (Marin, Contra Costa, San Francisco, Alameda, San Mateo, Santa Clara, Santa Cruz, Monterey, and San Benito), and is part of the National Cancer Institute’s Surveillance, Epidemiology, and End Results (SEER) Program and the California Cancer Registry (CCR). The GBACR region is home to more than 60 medical facilities that include a mix of community hospitals, academic centers, health maintenance organizations, Veterans’ Affairs (VA) hospitals, and independent physician practices. Eligible participants were those newly diagnosed with first primary breast or colorectal cancer (stages I–III) between 2019 and 2021, at least 18 years old, completed treatment, and resided in the GBACR catchment area at the time of diagnosis.

Using cancer registry case listing for recruitment allowed for oversampling of medically under-resourced populations. Medically under-resourced populations were defined as patients who were: non-Hispanic (NH) Black, Hispanic, or American Indian/Alaska Native; underinsured (uninsured or utilizing any non-Medicare public insurance); or living in low socioeconomic status (SES) neighborhoods defined as in the lower two quintiles within California. We aimed to recruit a study sample comprising approximately 40% breast cancer survivors, 30% female colorectal cancer survivors, and 30% male colorectal cancer survivors. Using the GBACR’s case listing mechanism, patients were identified approximately 12–18 months after diagnosis, an optimal time window to query about experiences regarding transition from active treatment to survivorship. Case listing data from GBACR can include data that is preliminary for recent diagnoses; updated data with cases complete through 2021 were later appended, and additional patients were excluded from the original recruitment pool based on updated tumor staging information.

**Fig. 1:**
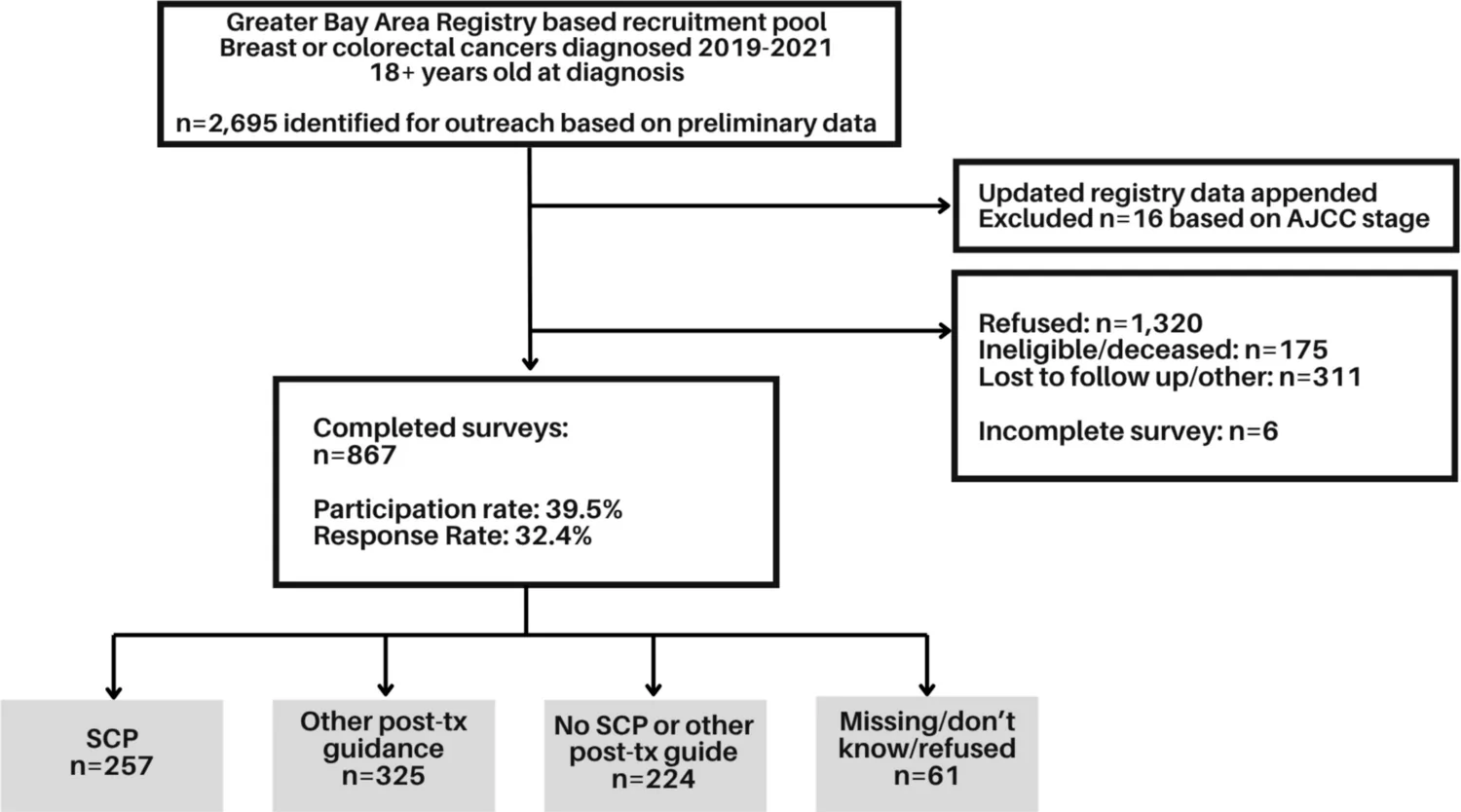
Flowchart of participant identification, eligibility, and recruitment for the CRUISE study, and classification by receipt/no receipt of survivorship care plan (SCP) or post-treatment guidance. Response rate is the proportion who responded among all those eligible (i.e., ineligible, deceased, and lost to follow-up were excluded from the denominator). Participation rate is the proportion who responded among all those contacted

Eligible participants were mailed study materials available in English, Spanish, and/or Chinese, reflecting the most common languages spoken in the catchment area. Participants completed the survey by telephone interview, mailed survey, or secured internet portal accessible from a computer or smartphone. Completion of the survey by telephone or portal took approximately 30 min. After completing the survey, participants were provided $40 in appreciation for their participation. Figure 1 shows participation and overall response rates. The study protocol was approved by institutional review boards at the University of California, San Francisco and the Committee for Human Subjects Protection for the state of California.

### Survey data

We assessed SCP or other post-treatment survivorship care guidance receipt using two questions: first we asked directly about SCP receipt, “Did anyone give you a survivorship care plan?” and if they did not say yes, we asked a follow-up question about receipt of post-treatment guidance, “Did a healthcare professional (i.e., doctor/nurse) ever provide you with guidelines or advice on managing your health after cancer treatment?”. In all analyses, responses to these two questions were combined as yes/no receipt of any SCP/post-treatment guidance. If a patient did not answer either question or responded that they did not know, they were categorized as missing and were excluded from analysis.

Sociodemographic factors collected in the CRUISE patient survey included race and ethnicity, relationship status, sex at birth, sexual orientation, education, employment, income adjusted for number of household members, home ownership, financial well-being, insurance type, nativity, language spoken at home, and social support [20]. Household income was collected using 10 categories and was operationalized for analysis by dividing the midpoint of each category by the number of people reported in the household. If the number of people was not reported in the household, income was classified as unknown.

Using guidelines from the American Cancer Society (ACS) for cancer survivors, information was collected on participants health behaviors, including fruit and vegetable intake, physical activity, smoking, and alcohol use [21]. Patient-reported outcomes included HRQOL, measured using FACT-G with four subscales (physical, social/family, emotional, and functional well-being) and the breast and colorectal cancer-specific domains, as well as comorbidities, and healthcare utilization [22].

### Cancer registry data

Clinical characteristics, including tumor characteristics (cancer site, AJCC stage, year of diagnosis), and type of reporting facility, were ascertained from the GBACR. Sociodemographic characteristics, including age, sex, race/ethnicity, and insurance type, and neighborhood socioeconomic status (nSES) [23] based on 2010 Census tracts and American Community Survey 2015–2019 data were used to define medically under-resourced status, assess representativeness of the study sample, and assess non-response biases. Lastly, data from the registry were used to supplement survey variables for sociodemographic characteristics when missing or unknown in the survey data (*n* = 6 for race and ethnicity, *n* = 4 for sex, *n* = 75 for insurance status, and *n* = 9 for relationship/marital status).

### Statistical analysis

To assess representativeness of survey respondents, we compared them to potentially eligible cases from the GBACR on sociodemographic and clinical characteristics from the cancer registry, and additionally stratified by medically under-resourced status. To assess non-response biases, we compared respondents to refusers on sociodemographic and clinical characteristics from the GBACR. Bivariate comparisons were conducted with t-tests and chi-square tests for continuous and categorical variables, respectively, and Bonferroni correction was applied to adjust the significance threshold from *α* = 0.05 to an adjusted *α* = 0.004 for Supplemental Tables 2 and 4 and an adjusted *α* = 0.005 for Supplemental Table 3.

We examined distributions of characteristics first across four SCP categories (received SCP, received post-treatment guidance, did not receive SCP/guidance, unknown), prior to combining the SCP and other post-treatment guidance groups and excluding those with missing responses (Supplemental Table 1). Comparisons across these four categories were conducted with ANOVA and chi-square tests. While we did not observe differences for most variables, differences were observed for sex, race/ethnicity, employment status, and financial well-being. After combining SCP and other post-treatment guideline responses  and excluding the missing responses, bivariate comparisons between those who did and did not receive SCPs/guidance were conducted with *t*-tests and chi-square tests.

Because the outcome of interest and receipt of SCPs/guidance were not rare, log binomial models were utilized for modeling univariable and multivariable associations between multilevel factors and receipt of SCPs/guidance. Relative risks (RRs) and 95% confidence intervals (CI) were calculated for unadjusted and fully adjusted models, with adjustment for clustering by census tract. For the multivariable model in the colorectal cancer subgroup only, the adjustment for clustering by census tract was removed to allow for model convergence. As breast cancer patients were all female, sex and cancer site were operationalized as a combined variable for use in the models including both cancer sites. *N* = 5 participants responded as insured but without any additional information; due to problems with model instability, these were collapsed into the referent group of “Insured: any private.” Variables were selected for inclusion in the fully adjusted models based on univariate associations where *p* < 0.1. We modeled associations among both cancer sites as well as among breast cancer and colorectal cancer survivors separately to explore if there were any cancer site-specific differences in receipt of SCPs/post-treatment guidance. In the colorectal cancer-specific model, those with unknown nativity were excluded due to small numbers causing model instability (*n* = 2).

Analyses were conducted in SAS 9.4 software for Windows (Cary, NC).

## Results

There were 2695 breast or colorectal cancer survivors, diagnosed between 2019 and 2021, identified for recruitment from the cancer registry (Fig. 1). Sixteen survivors were additionally excluded based on AJCC stage when updated data were appended. Study staff contacted 2679 survivors for the survey, of which 1812 refused, were ineligible, lost to follow up, or failed to complete a survey. In total, 867 survivors completed surveys for an overall response rate of 32.4%. Compared to registry eligible cases (*n* = 9684), survey participants (*n* = 867) were on average 1.8 years younger at diagnosis (Supplemental Table 2) and significantly different on every attribute examined except for marital status and the reporting facility type. Participants had a higher proportion of males (29.8% versus 18.2%), colorectal cancer diagnoses (58.7% vs. 32.9%) and survivors from medically under-resourced communities (65.2% and 34.9%, respectively) compared to registry eligible cases, which were an intentional result of the study sampling design. When limited to medically under-resourced patients (Supplemental Table 3), most of the differences were diminished; however, there were still more recent diagnoses (11.5% and 8.3% diagnosed in 2021, respectively) and AJCC stage III disease (26.7% and 21.0%, respectively) relative to registry eligible cases. All other characteristics were similar.

To examine response bias, sociodemographic characteristics between participants (*n* = 867) and non-participants (*n* = 1812) were compared (Supplemental Table 4). Study participants were on average 4.3 years younger at diagnosis, had a higher proportion of females (70.2% vs. 61.9%), were more likely to have a breast cancer diagnosis (41.3% vs. 31.3%), and were not recently diagnosed (10.7% vs. 13.1% diagnosed in 2021). Participants were also slightly less likely than non-participants to be from medically under-resourced communities (65.2% vs. 72.3%). Participants were more likely than non-participants to have been seen at a hospital that was an NCI-designated cancer center with an ACOS/COC cancer program (13.1% vs. 9.8%).

Out of 867 participants, 257 indicated they had received an SCP, 325 indicated they had received other post-treatment guidance, 224 indicated they had not received any guidance, and 61 did not provide information about whether they had received any guidance or not (Fig. 1). The group missing information was excluded from models, resulting in an analytical sample of *n* = 806.

Participants were 70.6% female and the mean age at diagnosis was 59 years old (Table 1). Approximately two-thirds (64.8%) of the participants were medically under-resourced, with 20.6% residing in low SES neighborhoods and 9.3% uninsured or on non-Medicare public insurance. Based on self-reported race and ethnicity, the study sample comprised 3.2% American Indian/Alaska Native, 18.6% Asian American/Pacific Islander, 25.2% Hispanic, 13.4% NH Black, and 38.0% NH White.

**Table 1:** Distributions of sociodemographic characteristics among breast and colorectal cancer survivors, by receipt/no receipt of survivorship guidance (either SCPs or other post-treatment guidance), diagnosed 2019–2021, Greater San Francisco Bay Area, CRUISE Study

	**Received guidance**	**No guidance**		**Overall**
	***N* (Row %)**^**1**^	***N* (Row %)**^**1**^	***p***	***N* (Col %)**^**1**^
**Total**	582 (72.2%)	224 (27.8%)		806 (100%)
**Age at diagnosis, mean (SD)**^**2**^	58.73 (13.44)	59.95 (14.22)	0.257	59.07 (13.66)
**Age at diagnosis categories**^**2**^				
<50	146 (74.5%)	50 (25.5%)	0.447	196 (24.3%)
50–59	163 (74.1%)	57 (25.9%)		220 (27.3%)
60–69	144 (72.0%)	56 (28.0%)		200 (24.8%)
70 +	129 (67.9%)	61 (32.1%)		190 (23.6%)
**Sex**^**3**^				
Male	187 (78.9%)	50 (21.1%)	**0.006**	237 (29.4%)
Female	395 (69.4%)	174 (30.6%)		569 (70.6%)
**Cancer site**^**3**^				
Breast cancer	227 (68.4%)	105 (31.6%)	**0.042**	332 (41.2%)
Colorectal cancer	355 (74.9%)	119 (25.1%)		474 (58.8%)
**AJCC stage**^**4**^				
I	277 (70.8%)	114 (29.2%)	0.194	391 (48.5%)
II	136 (69.7%)	59 (30.3%)		195 (24.2%)
III	169 (76.8%)	51 (23.2%)		220 (27.3%)
**Year of diagnosis**^**2**^				
2019	267 (73.2%)	98 (26.9%)	0.840	365 (45.3%)
2020	252 (71.2%)	102 (28.8%)		354 (43.9%)
2021	63 (72.4%)	24 (27.6%)		87 (10.8%)
**Medically under-resourced**^**2,4**^				
No	210 (73.9%)	74 (26.1%)	0.417	284 (35.2%)
Yes	372 (71.3%)	150 (28.7%)		522 (64.8%)
**Race/ethnicity group**^**3**^				
American Indian/Alaska Native	17 (65.4%)	<11	0.650	26 (3.2%)
Asian American/Pacific Islander	111 (74.0%)	39 (26.0%)		150 (18.6%)
Hispanic	148 (72.9%)	55 (27.1%)		203 (25.2%)
Non-Hispanic (NH) Black	80 (74.1%)	28 (25.9%)		108 (13.4%)
NH White	219 (71.6%)	87 (28.4%)		306 (38.0%)
Other/Multiple races/Unknown race	<11	<11		13 (1.6%)
**Insurance status**^**3**^				
Any Private	352 (73.8%)	125 (26.2%)	0.145	477 (59.2%)
Public only: Medicaid, Military, IHS, other	37 (63.8%)	21 (36.2%)		58 (7.2%)
Public only: Medicare	178 (70.1%)	76 (29.9%)		254 (31.5%)
Uninsured	15 (88.2%)	<11		17 (2.1%)
**Neighborhood SES (census tract) **^**2**^				
Q1 (lowest nSES)	31 (72.1%)	12 (27.9%)	0.699	43 (5.3%)
Q2	65 (74.7%)	22 (25.3%)		87 (10.8%)
Q3	86 (72.3%)	33 (27.7%)		119 (14.8%)
Q4	132 (68.8%)	60 (31.3%)		192 (23.8%)
Q5 (highest nSES)	241 (74.4%)	83 (25.6%)		324 (40.2%)
Unknown/Missing	27 (65.9%)	14 (34.2%)		41 (5.1%)
**Relationship status**^**3**^				
Partnered	384 (75.3%)	126 (24.7%)	**0.037**	510 (63.3%)
Unpartnered	192 (66.9%)	95 (33.1%)		287 (35.6%)
Other/Don’t know/Refused	<11	<11		<11
**Sexual orientation**^**5**^				
LGBTQIA +	63 (77.8%)	18 (22.2%)	0.490	81 (10.0%)
Straight/heterosexual	466 (71.7%)	184 (28.3%)		650 (80.6%)
Don’t know/Refused	53 (70.7%)	22 (29.3%)		75 (9.3%)
**Nativity**^**5**^				
Born in the United States	349 (72.7%)	131 (27.3%)	0.788	480 (59.6%)
Not born in the United States	228 (71.7%)	90 (28.3%)		318 (39.5%)
Don’t know/Refused	5 (62.5%	3 (37.5%)		8 (1.0%)
**Language spoken at home**^**5**^				
English only	354 (73.0%)	131 (27.0%)	0.597	485 (60.2%)
Language other than English	219 (70.7%)	91 (29.4%)		310 (38.5%)
Don’t know/Refused	9 (81.5%)	2 (18.2%)		11 (1.4%)
**Education**^**5**^				
High school education or below	156 (70.9%)	64 (29.1%)	0.737	220 (27.3%)
Some college, AA, or vocational/technical degree	164 (71.0%)	67 (29.0%)		231 (28.7%)
Bachelor’s, Master’s, doctoral, or professional degree	249 (73.5%)	90 (26.6%)		339 (42.1%)
Don’t know/Refused	13 (81.3%)	3 (18.8%)		16 (2.0%)
**Employment**^**5**^				
Employed	265 (79.6%)	68 (20.4%)	**0.001**	333 (41.3%)
Unemployed or on disability/leave	63 (61.8%)	39 (38.2%)		102 (12.7%)
Unpaid employment (homemaking, student, volunteer)	46 (64.8%)	25 (35.2%)		71 (8.8%)
Retired	187 (68.8%)	85 (31.3%)		272 (33.7%)
Don’t know/Refused	21 (75.0%)	7 (25.0%)		28 (3.5%)
**Income (adjusted for household size)**^**5**^				
Less than $25,000	155 (68.6%)	71 (31.4%)	0.651	226 (28.0%)
$25,000 to $54,999	126 (72.8%)	47 (27.2%)		173 (21.5%)
$55,000 to $99,999	123 (74.1%)	43 (25.9%)		166 (20.6%)
$100,000 and above	55 (76.4%)	17 (23.6%)		72 (8.9%)
Don’t know/refused	123 (72.8%)	46 (27.2%)		169 (21.0%)
**Home ownership**^**5**^				
Owned or being bought by you	315 (72.4%)	120 (27.6%)	0.698	435 (54.0%)
Rented for money	186 (70.7%)	77 (29.3%)		263 (32.6%)
Other/Don’t know/Refused	81 (75.0%)	27 (25.0%)		108 (13.4%)
**If you lost all your current income, you could live at your current address and standard of living**^**5**^				
Less than 1 month	63 (65.0%)	34 (35.1%)	0.360	97 (12.0%)
1–2 months	69 (70.4%)	29 (29.6%)		98 (12.2%)
3–6 months	75 (77.3%)	22 (22.7%)		97 (12.0%)
More than 6 months	233 (73.7%)	83 (26.3%)		316 (39.2%)
Don’t know/Refused	142 (71.7%)	56 (28.3%)		198 (24.6%)
**I feel confident that I can get medical care without being set back financially**^**5**^				
Strongly agree	232 (77.9%)	66 (22.2%)	**0.020**	298 (37.0%)
Agree	198 (70.7%)	82 (29.3%)		280 (34.7%)
Neither agree nor disagree	63 (60.0%)	42 (40.0%)		105 (13.0%)
Disagree	51 (75.0%)	17 (25.0%)		68 (8.4%)
Strongly disagree	27 (69.2%)	12 (30.8%)		39 (4.8%)
Don’t know/Refused	11 (68.8%)	5 (31.3%)		16 (2.0%)
**Reporting hospital type**^**2**^				
NCI-designated cancer center only	38 (69.1%)	17 (30.9%)	0.537	55 (6.8%)
NCI-designated cancer center + ACOS/COC cancer program	77 (72.0%)	30 (28.0%)		107 (13.3%)
ACOS/COC cancer program only	419 (73.4%)	152 (26.6%)		571 (70.8%)
Neither NCI nor ACOS/COC	48 (65.8%)	25 (34.3%)		73 (9.1%)

*AJCC*, American Joint Committee on Cancer; *ACOS*, American College of Surgeons; *COC*, Commission on Cancer; *NCI*, National Cancer Institute; bolded p-values indicate statistical significance with p-value < 0.05

^1^Per CCR guidelines, case counts <11 are suppressed for privacy in variables that utilized registry data

^2^Cancer registry or cancer registry derived variable

^3^Survey variable, supplemented with available cancer registry data

^4^Medically under-resourced patients were defined as being at least one of the following: Non-Hispanic Black, Hispanic, or American/Indian Alaska Native race or ethnicity as identified in the California Cancer Registry (CCR); underinsured (no insurance or utilizing any public insurance); living in neighborhoods with the lower 40% of socioeconomic status within California

^5^Survey variable

Based on unadjusted distributions, females were less likely to receive SCPs/guidance, hereafter guidance, (69.4% of females received guidance vs. 78.9% of males), as well as those who were unpartnered (66.9% of unpartnered people received guidance vs. 75.3% of partnered people). Those who were employed were more likely to receive guidance (79.6%), while those who were unemployed, retired, or had a form of unpaid work were less likely to receive guidance (61.8% of unemployed, 64.8% of unpaid work, 68.8% of retired). Those who received guidance were more likely to strongly agree that they could get the medical care they need without being set back financially. There were no differences for receipt of guidance by year of diagnosis, underserved status, nativity, education, household income, AJCC stage or facility type.

In unadjusted models including both cancer sites, breast cancer survivors (RR = 0.87, 95% CI: 0.79–0.96) and female colorectal cancer survivors (RR = 0.90, 95% CI: 0.81–1.00) were significantly less likely to receive guidance compared to male colorectal cancer survivors (Table 2). Compared to the partnered survivors, unpartnered survivors were less likely to receive guidance (RR = 0.89, 95% CI: 0.81–0.98). People who were unemployed, did unpaid work, or were retired were all less likely to have received survivorship guidance than those who were employed (RR = 0.78, 95% CI: 0.66–0.91 for unemployed; RR = 0.81, 95% CI: 0.68–0.98 for unpaid work; RR = 0.86, 95% CI: 0.79–0.95 for retired). Those who strongly agreed as well as those who disagreed with feeling financially secure were more likely to have received guidance than those who neither agreed nor disagreed (RR = 1.30, 95% CI: 1.10–1.53 for strongly agree; RR = 1.25, 95% CI: 1.03–1.51 for disagree).

**Table 2:** Unadjusted and adjusted risk ratios(RR) and 95% confidence intervals (CI) for associations between sociodemographic factors and receipt of survivorship guidance (SCPs as well as other types of survivorship guidance)

	Unadjusted RR (95% CI)	Adjusted RR (95% CI)
**Age category**^1^		
<50	Ref	
50–59	0.99 (0.88–1.12)	
60–69	0.97 (0.86–1.08)	
70+	0.91 (0.81–1.03)	
**Sex + cancer site**^**2**^		
CRC, male	Ref	Ref
CRC, female	**0.90 (0.81–1.00)**	0.93 (0.84–1.03)
Breast cancer, female	**0.87 (0.79–0.96)**	**0.89 (0.81–0.97)**
**AJCC Stage**		
I	Ref	
II	0.98 (0.87–1.11)	
III	1.08 (0.98–1.20)	
**Year of diagnosis**^**1**^		
2019	Ref	
2020	0.97 (0.89–1.07)	
2021	0.99 (0.85–1.15)	
**Medically under-resourced**^**1,**^^**4**^		
No	Ref	
Yes	0.96 (0.89–1.05)	
**Race/ethnicity**^**2**^		
American Indian/Alaska Native	0.91 (0.69–1.21)	
Asian American/Pacific Islander	1.03 (0.92–1.16)	
Hispanic	1.02 (0.91–1.14)	
NH Black	1.04 (0.91–1.18)	
NH White	Ref	
Other/multiple race/unknown	0.75 (0.45–1.25)	
**Insurance status**^**2**^		
Any private	Ref	
Public only: Medicaid, Military, IHS, Other	0.86 (0.71–1.06)	
Public only: Medicare	0.95 (0.85–1.06)	
Uninsured	1.20 (1.00–1.43)	
**Neighborhood SES (census tract)**^1^		
Q1 (lowest nSES)	Ref	
Q2	1.04 (0.85–1.27)	
Q3	1.00 (0.82–1.22)	
Q4	0.95 (0.79–1.15)	
Q5 (highest nSES)	1.03 (0.86–1.23)	
Missing	0.91 (0.77–1.08)	
**Relationship status**^**3**^		
Partnered	Ref	Ref
Unpartnered	**0.89 (0.81–0.98)**	0.93 (0.86–1.02)
Don’t know/refused	0.89 (0.56–1.41)	0.89 (0.56–1.41)
**Sexual orientation**^**3**^		
LGBTQIA+	1.08 (0.95–1.23)	
Straight/heterosexual	Ref	
Don’t know	0.99 (0.84–1.15)	
**Nativity**^**3**^		
Born in the United States	Ref	
Not born in the United States	0.99 (0.90–1.07)	
Don’t know/refused	0.86 (0.50–1.47)	
**Language spoken at home**^**3**^		
English	Ref	
Language other than English	0.97 (0.89–1.05)	
Don’t know/refused	1.12 (0.84–1.49)	
**Education**^**3**^		
High school education or below	Ref	
Some college/AA/vocational	1.00 (0.89–1.12)	
Bachelor’s and above	1.04 (0.93–1.15)	
Refused	1.15 (0.89–1.47)	
**Employment**^**3**^		
Employed	Ref	Ref
Unemployed	**0.78 (0.66–0.91)**	**0.80 (0.68–0.94)**
Unpaid employment (homemaking, student, volunteer)	**0.81 (0.68–0.98)**	0.84 (0.70–1.00)
Retired	**0.86 (0.79–0.95)**	**0.85 (0.77–0.95)**
Refused	0.94 (0.75–1.18)	1.08 (0.87–1.35)
**Income (adjusted for household size)**^**3**^		
Less than $25,000	0.90 (0.77–1.04)	
$25,000 to $54,999	0.95 (0.82–1.11)	
$55,000 to $99,999	0.97 (0.83–1.13)	
$100,000 or more	Ref	
Unknown/Refused	0.95 (0.82–1.11)	
**Home ownership**^**3**^		
Owned or bought	Ref	
Rented for money	0.98 (0.89–1.07)	
Other/Don’t know/Refused	1.04 (0.92–1.16)	
**If you lost all your current income, you could live at your current address and standard of living**		
Less than 1 month	0.88 (0.75–1.04)	
1–2 months	0.95 (0.82–1.11)	
3–6 months	1.05 (0.92–1.19)	
More than 6 months	Ref	
Unknown/Refused	0.97 (0.87–1.09)	
**I feel confident that I can get medical care without being set back financially**^**3**^		
Strongly agree	**1.30 (1.10–1.53)**	**1.28 (1.09–1.51)**
Agree	1.18 (0.99–1.41)	1.16 (0.97–1.38)
Neither agree nor disagree	Ref	Ref
Disagree	**1.25 (1.03–1.51)**	**1.22 (1.01–1.46)**
Strongly disagree	1.15 (0.89–1.50)	1.17 (0.92–1.49)
Unknown/Refused	1.15 (0.80–1.64)	1.14 (0.80–1.61)
**Hospital type**^1^		
Neither NCI nor ACOS/COC	Ref	
NCI-designated cancer center +ACOS/COC cancer program	1.09 (0.89–1.35)	
NCI-designated cancer center only	1.05 (0.82–1.34)	
ACOS/COC cancer program only	1.12 (0.93–1.34)	

*AJCC*, American Joint Committee on Cancer; *ACOS*, American College of Surgeons; *COC*, Commission on Cancer; *NCI*, National Cancer Institute

Models with univariate associations *p* < 0.1 were included in an adjusted model. Variables that were included in the adjusted model are shown in the Adjusted RR column. Bolded associations are statistically significant at *p* < 0.05

^1^Cancer registry or cancer registry-derived variable

^2^Survey variable, supplemented with available cancer registry data

^3^Survey variable

^4^Medically under-resourced patients were defined as being at least one of the following: Non-Hispanic Black, Hispanic, or American/Indian Alaska Native race or ethnicity as identified in CCR; underinsured (no insurance or utilizing any public insurance); living in neighborhoods with the lower 40% of socioeconomic status within California

The adjusted model included sex+cancer site, relationship status, employment status, and financial well-being. The effect estimates were minimally different than in the unadjusted models; female breast cancer survivors (RR = 0.89, 95% CI 0.81–0.97), unemployed (RR = 0.80, 95% CI 0.68–0.94), and retired (RR = 0.85, 95% CI 0.77–0.95) people remained significantly less likely to receive guidance. Those who strongly agreed as well as those who disagreed that they could get medical care without financial setbacks were also significantly more likely to have received guidance than those who neither agreed nor disagreed (RR = 1.28, 95% CI 1.09–1.51 for strongly agree; RR = 1.22, 95% CI 1.01–1.46 for disagree).

Among breast cancer survivors, unemployment (RR = 0.70, 95% CI 0.50–1.00), being retired (RR = 0.81, 95% CI 0.68–0.96), having low income (less than $25,000; RR = 0.76, 95% CI 0.59–0.99), and renting a home (RR = 0.79, 95% CI 0.66–0.95) were significantly associated with lower likelihood of receiving guidance in the unadjusted models (Table 3). High confidence in being able to receive medical care without financial setbacks was significantly associated with higher likelihood of receiving guidance (RR = 1.35, 95% CI: 1.03–1.78). In the adjusted model, employment status, income, and home ownership were included in the model, and being retired (RR = 0.81, 95% CI 0.68–0.96) and renting a home (RR = 0.81, 95% CI 0.67–0.98) remained significantly associated with receiving guidance.

**Table 3:** Unadjusted and adjusted risk ratios (RR) and 95% confidence intervals (CI) for associations between sociodemographic factors and receipt of survivorship guidance (SCPs as well as other types of survivorship guidance) among breast cancer survivors

	**N**^**1**^	**Unadjusted RR (95% CI)**	**Adjusted RR (95% CI)**
**Total**	**332**		
**Age at diagnosis category**^**2**^			
<50	98	Ref	
50–59	92	1.02 (0.84–1.23)	
60–69	81	0.98 (0.81–1.19)	
70+	61	0.92 (0.74–1.15)	
**AJCC stage**^**2**^			
I	250	Ref	
II	57	0.98 (0.87–1.11)	
III	25	1.08 (0.98–1.20)	
**Year of diagnosis**^**2**^			
2019	180	Ref	
2020	152	0.95 (0.82–1.10)	
**Medically under-resourced**^**2,3**^			
No	101	Ref	
Yes	231	0.94 (0.81–1.10)	
**Race/ethnicity**^**4**^			
American Indian/Alaska Native	<11	0.80 (0.44–1.46)	
Asian American/Pacific Islander	63	0.98 (0.80–1.20)	
Hispanic	85	0.95 (0.78–1.15)	
NH Black	55	1.05 (0.84–1.30)	
NH White	115	Ref	
Other/multiple races/unknown race	<11	0.86 (0.41–1.81)	
**Insurance**^**4**^			
Any Private	202	Ref	
Public only: Medicaid, Military, IHS, Other	29	0.82 (0.60–1.13)	
Public only: Medicare	94	0.90 (0.74–1.09)	
Uninsured	<11	1.20 (0.88–1.65)	
**Neighborhood SES (census tract) **^**2**^			
Q1 (lowest nSES)	43	Ref	
Q2	87	1.23 (0.78–1.94)	
Q3	119	1.06 (0.66–1.70)	
Q4	192	1.14 (0.73–1.78)	
Q5 (highest nSES)	324	1.22 (0.79–1.87)	
Missing	41	1.07 (0.70–1.61)	
**Relationship status**^**5**^			
Partnered	199	Ref	
Other/don’t know/refused	<11	1.05 (0.59–1.86)	
Unpartnered	129	0.89 (0.76–1.04)	
**Sexual orientation**^**5**^			
LGBTQIA+	33	1.11 (0.90–1.37)	
Straight/heterosexual	266	Ref	
Don’t know/refused	33	0.94 (0.71–1.23)	
**Nativity**^**5**^			
Born in the United States	187	Ref	
Not born in the United States	139	0.92 (0.79–1.07)	
Don’t know/refused	6	1.18 (0.81–1.72)	
**Language spoken at home**^**5**^			
English	188	Ref	
Language other than English	141	0.94 (0.81–1.09)	
Don’t know/refused	3	0.95 (0.42–2.13)	
**Education**^**5**^			
High school education or below	92		
Some college, AA, or vocational/technical degree	86	1.03 (0.84–1.27)	
Bachelor’s, Master’s, doctoral, or professional degree	147	1.08 (0.91–1.30)	
Don’t know/refused	7	1.10 (0.67–1.80)	
**Employment**^**5**^			
Employed	153	Ref	Ref
Unemployed or on disability/leave	30	**0.70 (0.50–1.00)**	0.77 (0.55–1.07)
Unpaid employment (homemaking, student, volunteer)	44	0.87 (0.69–1.09)	0.90 (0.70–1.15)
Retired	91	**0.81 (0.68–0.97)**	**0.81 (0.68–0.96)**
Don’t know/refused	14	0.94 (0.66–1.34)	1.01 (0.73–1.40)
**Income (adjusted for household size)**^**5**^			
Less than $25,000	90	**0.76 (0.59–0.99)**	0.88 (0.68–1.13)
$25,000 to $54,999	65	1.00 (0.79–1.26)	1.02 (0.82–1.26)
$55,000 to $99,999	70	0.87 (0.67–1.13)	0.89 (0.70–1.13)
$100,000 and above	28	Ref	Ref
Unknown/Refused	79	0.84 (0.65–1.07)	0.90 (0.69–1.18)
**Home ownership**^**5**^			
Owned or bought	191	Ref	Ref
Rented for money	104	**0.79 (0.66–0.95)**	**0.81 (0.67–0.98)**
Other/Don’t know/Refused	37	1.04 (0.84–1.29)	0.90 (0.69–1.18)
**If you lost all your current income, you could live at your current address and standard of living**^**5**^			
Less than 1 month	44	0.80 (0.60–1.05)	
1–2 months	39	0.90 (0.69–1.16)	
3–6 months	40	0.94 (0.76–1.18)	
More than 6 months	128	Ref	
Unknown/Refused	81	0.86 (0.72–1.05)	
**I feel confident that I can get medical care without being set back financially**^**5**^			
Strongly agree	117	**1.35 (1.03–1.78)**	
Agree	118	1.18 (0.89–1.55)	
Neither agree nor disagree	48	Ref	
Disagree	30	1.30 (0.93–1.82)	
Strongly disagree	15	1.07 (0.66–1.72)	
Unknown/Refused	4	0.89 (0.33–2.40)	
**Hospital type**^**2**^			
Neither NCI nor ACOS/COC	20	Ref	
NCI-designated cancer center+ACOS/COC cancer program	53	0.94 (0.61–1.47)	
NCI-designated cancer center only	26	1.22 (0.78–1.89)	
ACOS/COC cancer program only	233	1.19 (0.81–1.74)	

*AJCC*, American Joint Committee on Cancer; *ACOS*, American College of Surgeons; *COC*, Commission on Cancer; *NCI*, National Cancer Institute

Models with univariate associations *p* < 0.1 were included in an adjusted model. Variables that were included in the adjusted model are shown in the Adjusted RR column. Bolded associations are statistically significant at *p* < 0.05

^1^Per CCR guidelines, case counts <11 are suppressed for privacy in variables that utilized registry data

^2^Cancer registry or cancer registry derived variable

^3^Medically under-resourced patients were defined as being at least one of the following: Non-Hispanic Black, Hispanic, or American/Indian Alaska Native race or ethnicity as identified in CCR; underinsured (no insurance or utilizing any public insurance); living in neighborhoods with the lower 40% of socioeconomic status within California

^4^Survey variable, supplemented with available cancer registry data

^5^Survey variable

Among colorectal cancer survivors, female participants (RR = 0.90, 95% CI 0.81–1.00), missing nSES (RR = 0.84, 95% CI 0.73–0.98), unemployment (RR = 0.79, 95% CI 0.66–0.94), and retirement (RR = 0.87, 95% CI 0.79–0.97) were significantly associated with lower likelihood of receiving guidance. Those who strongly agreed with being financially secure were more likely to receive guidance (RR = 1.25, 95% CI 1.01–1.54), as was having been seen at an NCI-designated cancer center with an ACOS/COC cancer program (RR = 1.28, 95% CI 1.04–1.58). Sex, employment status, and hospital type were included in the adjusted model. Effect estimates were minimally different in the adjusted model, and unemployment (RR = 0.79, 95% CI 0.66–0.94), retirement (RR = 0.89, 95% CI 0.79–0.99), and being seen at an NCI-designated cancer center with an ACOS/COC cancer program (RR = 1.27, 95% CI 1.01–1.6) remained significant associations (Table 4).

**Table 4:** Unadjusted and adjusted risk ratios (RR) and 95% confidence intervals (CI) for associations between sociodemographic factors and receipt of survivorship guidance (SCPs as well as other types of survivorship guidance) among colorectal cancer survivors

	*N*^1^	Unadjusted RR (95% CI)	Adjusted RR (95% CI)
**Total**	**474**		
**Sex**^**2**^			
Male	237	Ref	Ref
Female	237	**0.90 (0.81–1.00)**	0.92 (0.83–1.03)
**Age category**^**3**^			
<50	98	Ref	
50–59	128	0.96 (0.84–1.11)	
60–69	119	0.94 (0.82–1.08)	
70+	129	0.88 (0.76–1.01)	
**AJCC stage**^**3**^			
I	141	Ref	
II	138	0.98 (0.87–1.11)	
III	195	1.08 (0.98–1.20)	
**Year of diagnosis**^**3**^			
2019	185	Ref	
2020	202	0.98 (0.88–1.09)	
2021	87	0.95 (0.81–1.12)	
**Medically under-resourced**^**3,4**^			
No	183	Ref	
Yes	291	0.99 (0.89–1.09)	
**Race/ethnicity**^**2**^			
American Indian/Alaska Native	17	0.97 (0.70–1.33)	
Asian American/Pacific Islander	87	1.07 (0.93–1.24)	
Hispanic	118	1.07 (0.94–1.22)	
NH Black	53	1.04 (0.88–1.22)	
NH White	191	Ref	
Other/multiple races/unknown race	<11	0.69 (0.34–1.38)	
**Insurance**^**2**^			
Any private	275	Ref	
Public only: Medicaid, Military, IHS, Other	29	0.90 (0.71–1.16)	
Public only: Medicare	160	0.97 (0.86–1.08)	
Uninsured	<11	1.18 (0.95–1.45)	
**Neighborhood SES (census tract) **^**3**^			
Q1 (lowest nSES)	26	Ref	
Q2	47	0.95 (0.77–1.17)	
Q3	74	0.97 (0.80–1.17)	
Q4	119	0.86 (0.72–1.04)	
Q5 (highest nSES)	183	0.95 (0.80–1.12)	
Missing	25	**0.84 (0.73–0.98)**	
**Relationship status**^**2**^			
Partnered	311	Ref	
Unpartnered	158	0.89 (0.80–1.00)	
Other/Refused/Missing	< 11	0.77 (0.38–1.58)	
**Sexual orientation**^**5**^			
LGBTQIA+	48	1.07 (0.91–1.25)	
Straight/heterosexual	384	Ref	
Don’t know/refused	42	1.03 (0.86–1.23)	
**Nativity**^**5**^			
Born in the United States	293	Ref	
Not born in the United States	179	1.04 (0.94–1.15)	
**Language spoken at home**^**5**^			
English	297	Ref	
Language other than English	169	1.00 (0.90–1.11)	
Don’t know/refused	8	1.17 (0.89–1.53)	
**Education**^**5**^			
High school education or below	128		
Some college, AA, or vocational/technical degree	145	0.97 (0.85–1.11)	
Bachelor’s, Master’s, doctoral, or professional degree	192	1.01 (0.89–1.14)	
Don’t know/refused	9	1.19 (0.92–1.52)	
**Employment**^**5**^			
Employed	180	Ref	Ref
Unemployed	72	**0.79 (0.66–0.94)**	**0.79 (0.66–0.94)**
Unpaid employment (homemaking, student, volunteer)	27	0.76 (0.57–1.02)	0.81 (0.61–1.07)
Retired	181	**0.87 (0.79–0.97)**	**0.89 (0.79–0.99)**
Refused	14	0.95 (0.72–1.26)	0.88 (0.67–1.15)
**Income (adjusted for household size)**^**5**^			
Less than $25,000	136	0.99 (0.82–1.19)	
$25,000 to $54,999	108	0.93 (0.76–1.13)	
$55,000 to $99,999	96	1.04 (0.86–1.27)	
$100,000 and above	44	Ref	
Unknown/Refused	90	1.05 (0.87–1.28)	
**Home ownership**^**5**^			
Owned or bought	244	Ref	
Rented for money	159	1.10 (0.99–1.22)	
Other/Don’t know/Refused	71	1.03 (0.90–1.20)	
**If you lost all your current income, you could live at your current address and standard of living: **^**5**^			
Less than 1 month	53	0.95 (0.78–1.15)	
1–2 months	59	0.99 (0.83–1.19)	
3–6 months	57	1.12 (0.96–1.31)	
More than 6 months	188	Ref	
Unknown/Refused	117	1.05 (0.92–1.19)	
**I feel confident that I can get medical care without being set back financially: **^**5**^			
Strongly agree	181	**1.25 (1.01–1.54)**	
Agree	162	1.17 (0.94–1.46)	
Neither agree nor disagree	57	Ref	
Disagree	38	1.21 (0.95–1.54)	
Strongly disagree	24	1.19 (0.88–1.61)	
Unknown/Refused	12	1.19 (0.81–1.74)	
**Hospital type**^**3**^			
Neither NCI nor ACOS/COC	53	Ref	Ref
NCI-designated cancer center+ACOS/COC cancer program	54	**1.28 (1.04–1.58)**	**1.27 (1.01–1.60)**
NCI-designated cancer center only	29	0.96 (0.71–1.32)	0.98 (0.73–1.30)
AOCS/COC cancer program only	338	1.10 (0.91–1.34)	1.10 (0.92–1.31)

*AJCC*, American Joint Committee on Cancer; *ACOS*, American College of Surgeons; *COC*, Commission on Cancer; *NCI*, National Cancer Institute

Models with univariate associations *p* < 0.1 were included in an adjusted model. Variables that were included in the adjusted model are shown in the Adjusted RR column. Bolded associations are statistically significant at *p* < 0.05

^1^Per CCR guidelines, case counts < 11 are suppressed for privacy in variables that utilized registry data

^2^Survey variable, supplemented with available cancer registry data

^3^Cancer registry or cancer registry derived variable

^4^Medically under-resourced patients were defined as being at least one of the following: Non-Hispanic Black, Hispanic, or American/Indian Alaska Native race or ethnicity as identified in CCR; underinsured (no insurance or utilizing any public insurance); living in neighborhoods with the lower 40% of socioeconomic status within California

^5^Survey variable

## Discussion

While population-based cancer registries have been used in research studies for recruitment of cancer survivors, the CRUISE Study is the first to use this approach to understand multilevel factors associated with survivorship care plan/post-treatment guidance receipt across diverse populations. Leveraging sociodemographic information available from cancer registries, we were additionally able to oversample patients from medically under-resourced communities, to ensure their adequate representation, and address a key gap in the literature [15]. This approach allowed us to identify differences across clinical practices beyond sociodemographic factors. The survivors included in this study represent a wide range of treatment histories and life experiences, with a diverse set of survivorship needs. Deliberate oversampling of the medically under-resourced population provides insight into the survivorship experiences of patients who are often overlooked.

While we did not find that race/ethnicity and medically under-resourced status were associated with survivorship care plan/guidance receipt, we did find several other sociodemographic factors associated with lower likelihood of receipt, including unemployed or retired status among breast and colorectal cancer patients, while confidence in being able to receive medical care without financial setbacks was associated with higher likelihood of receipt. Home rentership versus ownership was also associated with higher likelihood of receipt among breast cancer survivors. Some of these associations suggest that lower SES is associated with less frequent receipt of survivorship care guidance, as reported in a scoping review of social determinants of health and cancer survivorship [24]. However, other associations in our sample show higher likelihood of receipt among those traditionally considered to be medically under-resourced, counter to our hypothesis. Also unexpected in our results are the lack of differences in SCP/guidance receipt across racial/ethnic groups. These associations may also illustrate the complexity of issuance of SCPs or survivorship guidance, given the evolving medical recommendations [14, 25] at the time our participants were completing active treatment, lack of tailored content in these plans, and inconsistent implementation across medical settings given lack of clarity around which medical professional is responsible for the issuance [26]. In addition, patients may not remember that they have received an SCP or survivorship care guidance.

The CRUISE study demonstrated that it is feasible to recruit cancer survivors from population-based cancer registries with over-representation of medically under-resourced populations (64%). In particular, relative to registry-eligible cases, we were able to overrepresent NH Black survivors (13% sample, 6% cancer registry, 5% general CA population), Hispanic survivors (29% sample, 15% registry, 39% CA), survivors with non-Medicare public insurance (46% sample, 42% registry, 38% CA), and survivors residing in low SES neighborhoods (21% sample, 12% registry, 39% CA). We were successful in achieving this overrepresentation as our study design focused on oversampling these population groups, made the study available in other languages than English that are commonly spoken in our areas, and followed up with telephone calls to ensure participants had received our mail invitation and fielded questions to address any concerns in participation. Future studies can leverage similar strategies to engage diverse cancer survivors to better understand their survivorship experiences as well as the multilevel factors that contribute to outcomes, as well as health disparities faced by this growing population.

In this relatively contemporary cancer survivor cohort, we found a high prevalence of receipt of SCPs/guidance, with receipt by 68% of breast cancer survivors, 71% of female colorectal cancer survivors, and 79% of male colorectal cancer survivors. Females were slightly less likely than males, and breast cancer patients were less likely than colorectal cancer patients, to have received a plan, which contrasts with prior research [27]. These overall percentages of patient reporting having received an SCP/guidance are higher than the percentages reported in the 2024 National Survey of Cancer Patients, Survivors, and Caregivers, where 43% of patients overall, 58% in urban settings, reported having received a plan [28]. A sizable proportion of patients (90%) in our study were seen at either an NCI-designated cancer center (20%) and/or an ACoS or CoC accredited facility (84%), where SCPs were required for ACoS/COC accreditation between 2012–2019, and although not required for NCI cancer center designation, they are broadly used across cancer centers [29]. However, the aforementioned national survey found that while most patients reported receiving information about treatments received, tumor types, surveillance/screening schedule, and expected side effects, much fewer patients reported receiving other types of information, such as on health behaviors, and psychosocial side effects and resources for mitigation [28], indicating heterogeneity in SCP content provided across facilities. A limitation of our study is the inability to assess variability in patient experiences within facilities.

There are several additional limitations in the CRUISE Study that should be noted. Our response and participation rates are low relative to recruitment studies of cancer patients and survivors in clinical settings but are consistent with contemporary population-based studies recruiting from cancer registries [30]. Nonetheless, we have demonstrated feasibility of our approach to use an established population-based cancer registry and available sociodemographic information to yield a study sample that overrepresents medically under-resourced patients. Our approach of recruiting from a defined source population also allowed us to assess and quantify the degree of non-representativeness and non-response bias. Although we relied on patient report and recall to determine the receipt of SCPs, this outcome is nonetheless relevant as reflective of patients’ experiences. Finally, although the study sample is population-based, it is set in one geographic area and thus these results may not be generalizable to other areas of the US.

The CRUISE Study demonstrated feasibility in working with a cancer registry to accrue a population-based sample of cancer survivors with over-representation of medically under-resourced patients. While we found that a high proportion received survivorship guidance, including survivorship care plans or post-treatment guidance, variations were noted across some patient characteristics. Further research is needed to assess whether receipt of survivorship guidance is associated with improved patient outcomes.

## Supplementary Information

Below is the link to the electronic supplementary material.

ESM1 (DOCX 60.4 KB)

## Data Availability

The data used in this manuscript are available upon request and approval from the CRUISE Principal Investigators (U. Sarkar & S. Lin Gomez) and from the California Cancer Registry.
