# Supplementary material for: Survivorship care guidance in a diverse cancer survivor population: Cancer Registry for Understanding and Improving Survivorship Experiences (CRUISE) Study
Source: J Cancer Surviv. 2026 Jan 28;20(Suppl 2):1450–66. doi: 10.1007/s11764-026-01970-1 (PMC13744537; doi:10.1007/s11764-026-01970-1)
Supplement: Supplementary file 1 [file 11764_2026_1970_MOESM1_ESM.docx]

**SUPPLEMENTAL TABLES**

Supplemental Table 1: Distributions of sociodemographic variables among breast and colorectal cancer survivors, by receipt/no receipt of survivorship care plan (SCP) or post-treatment guidance, diagnosed 2019-2021, Greater San Francisco Bay Area, CRUISE Study

|  | **SCP** | **Other Post-Treatment Guidance** | **No SCP or Post-Treatment Guidance** | **Missing SCP/Guide Response** |  | **Overall** |
| --- | --- | --- | --- | --- | --- | --- |
|  | **N (Row %)^1^** | **N (Row %)^1^** | **N (Row %)^1^** | **N (Row %)^1^** | **p** | **N (Col %)^1^** |
| **Total** | 257 (29.6%) | 325 (37.5%) | 224 (25.8%) | 61 (7.0%) |  | 867 (100%) |
| **Age at diagnosis, mean (SD) ^2^** | 58.18 (12.84) | 59.17 (13.89) | 59.95 (14.22) | 58.03 (15.32) | 0.505 | 59.00 (13.78) |
| **Age categories^2^** |  |  |  |  |  |  |
| 24-49 | 66 (30.8%) | 80 (37.4%) | 50 (23.4%) | 18 (8.4%) | 0.712 | 214 (24.7%) |
| 50-59 | 75 (32.1%) | 88 (37.6%) | 57 (24.4%) | 14 (6.0%) |  | 234 (27.0%) |
| 60-69 | 67 (31.3%) | 77 (36.0%) | 56 (26.2%) | 14 (6.5%) |  | 214 (24.7%) |
| 70+ | 49 (23.9%) | 80 (39.0%) | 61 (29.8%) | 15 (7.3%) |  | 205 (23.6%) |
| **Sex^3^** |  |  |  |  |  |  |
| Male | 84 (32.6%) | 103 (39.9%) | 50 (19.4%) | 21 (8.1%) | **0.042** | 258 (29.8%) |
| Female | 173 (28.4%) | 222 (36.5%) | 174 (28.6%) | 40 (6.6%) |  | 609 (70.2%) |
| **Cancer type^3^** |  |  |  |  |  |  |
| Breast cancer | 107 (28.9%) | 120 (33.5%) | 105 (29.3%) | 26 (7.3%) | 0.139 | 358 (29.8%) |
| Colorectal cancer | 150 (29.5%) | 205 (40.3%) | 119 (23.4%) | 35 (6.9%) |  | 509 (58.7%) |
| **AJCC stage^2^** |  |  |  |  |  |  |
| I | 121 (28.6%) | 156 (36.9%) | 114 (27.0%) | 32 (7.6%) | 0.711 | 423 (48.8%) |
| II | 60 (28.7%) | 76 (36.4%) | 59 (28.2%) | 14 (6.7%) |  | 209 (24.1%) |
| III | 76 (32.3%) | 93 (39.6%) | 51 (21.7%) | 15 (6.4%) |  | 235 (27.1%) |
| **Year of diagnosis^2^** |  |  |  |  |  |  |
| 2019 | 112 (28.3%) | 155 (39.1%) | 98 (24.8%) | 31 (7.8%) | 0.910 | 396 (45.7%) |
| 2020 | 117 (31.0%) | 135 (35.7%) | 102 (27.0%) | 24 (6.4%) |  | 378 (43.6%) |
| 2021 | 28 (30.1%) | 35 (37.6%) | 24 (25.8%) | <11 |  | 93 (10.7%) |
| **Medically under-resourced^4^** |  |  |  |  |  |  |
| No | 84 (27.8%) | 126 (41.7%) | 74 (24.5%) | 18 (6.0%) | 0.281 | 302 (34.8%) |
| Yes | 173 (30.6%) | 199 (35.2%) | 150 (26.6%) | 43 (7.6%) |  | 565 (65.2%) |
| **Race/ethnicity group^3^** |  |  |  |  |  |  |
| American Indian/Alaska Native | <11 | <11 | <11 | 0 (0.0%) | **0.015** | 26 (3.0%) |
| Asian American/Pacific Islander | 48 (28.6%) | 63 (37.5%) | 39 (23.2%) | 18 (10.7%) |  | 168 (19.4%) |
| Hispanic | 81 (37.3%) | 67 (30.9%) | 55 (25.4%) | 14 (6.5%) |  | 217 (25.0%) |
| NH Black | 33 (28.5%) | 47 (40.5%) | 28 (24.1%) | <11 |  | 116 (13.4%) |
| NH White | 82 (25.4%) | 137 (42.4%) | 87 (26.9%) | 17 (5.3%) |  | 323 (37.3%) |
| Unknown/Missing | <11 | <11 | <11 | <11 |  | 17 (2.0%) |
| **Insurance status^3^** |  |  |  |  |  |  |
| Any Private | 155 (30.4%) | 197 (38.6%) | 125 (24.5%) | 33 (6.5%) | 0.198 | 510 (58.8%) |
| Public only: Medicare, Military, IHS, other | 12 (18.2%) | 25 (37.9%) | 21 (31.8%) | <11 |  | 66 (7.6%) |
| Public only: Medicare | 82 (29.9%) | 96 (35.0%) | 76 (27.7%) | 20 (7.3%) |  | 274 (31.6%) |
| Uninsured | <11) | <11 | <11 | 0 (0.0%) |  | 17 (2.0%) |
| **Neighborhood SES (census tract) ^2^** |  |  |  |  |  |  |
| Q1 (lowest nSES) | 10 (22.7%) | 21 (47.7%) | 12 (27.3%) | 1 (2.3%) | 0.614 | 44 (5.1%) |
| Q2 | 27 (28.1%) | 38 (39.6%) | 22 (22.9%) | 9 (9.4%) |  | 96 (11.1%) |
| Q3 | 42 (32.1%) | 44 (33.6%) | 33 (25.2%) | 12 (9.2%) |  | 131 (15.1%) |
| Q4 | 64 (31.2%) | 68 (33.2%) | 60 (29.3%) | 13 (6.3%) |  | 205 (23.6%) |
| Q5 (highest nSES) | 99 (28.5%) | 142 (40.8%) | 83 (23.9%) | 24 (6.9%) |  | 348 (40.1% |
| Unknown/Missing | 15 (34.9%) | 12 (27.9%) | 14 (32.6%) | 2 (4.6%) |  | 43 (5.0%) |
| **Relationship status^3^** |  |  |  |  |  |  |
| Single | 30 (25.9%) | 43 (37.1%) | 35 (30.2%) | 8 (6.9%) | 0.188 | 116 (13.4%) |
| Legally married / registered domestic partners | 148 (32.0%) | 175 (37.8%) | 103 (22.3%) | 37 (8.0%) |  | 463 (53.4%) |
| Separated | <11 | <11 | <11 | <11 |  | 27 (3.1%) |
| Divorced | 24 (29.6%) | 26 (32.1%) | 28 (34.6%) | <11 |  | 81 (9.3%) |
| Widowed | 19 (24.1%) | 33 (41.8%) | 24 (30.4%) | <11 |  | 79 (9.1%) |
| In a relationship but not living with partner | 12 (38.7%) | 16 (51.6%) | <11 | 0 (0.0%) |  | 31 (3.6%) |
| Living with a partner to whom you are not married | 12 (20.0%) | 21 (35.0%) | 20 (33.3%) | <11 |  | 60 (6.9%) |
| Other/Don't know/Refused | <11 | <11 | <11 | <11 |  | <11 |
| **Relationship status (simplified)** **^3^** |  |  |  |  |  |  |
| Partnered | 172 (31.1%) | 212 (38.3%) | 126 (22.7%) | 44 (7.9%) | 0.163 | 554 (63.9%) |
| Unpartnered | 82 (27.1%) | 110 (36.3%) | 95 (31.4%) | 16 (5.3%) |  | 303 (34.9%) |
| Other/Refused/Missing | <11 | <11 | <11 | <11 |  | <11 |
| **Sexual orientation^5^** |  |  |  |  |  |  |
| LGBTQIA+ | 27 (30.3%) | 36 (40.5%) | 18 (20.2%) | <11 | 0.644 | 89 (10.3%) |
| Straight/heterosexual | 206 (29.7%) | 260 (37.5%) | 184 (26.5%) | 44 (6.3%) |  | 694 (80.0%) |
| Don't know/Refused | 24 (28.6%) | 29 (34.5%) | 22 (26.2%) | 9 (10.7%) |  | 84 (9.7%) |
| **Nativity^5^** |  |  |  |  |  |  |
| Born in the United States | 144 (28.1%) | 205 (40.0%) | 131 (25.6%) | 32 (6.3%) | 0.456 | 512 (59.1%) |
| Not born in the United States | 110 (31.7%) | 118 (34.0%) | 90 (25.9%) | 29 (8.4%) |  | 347 (40.0%) |
| Don't know/Refused | 3 (37.5%) | 2 (25.0%) | 3 (37.5%) | 0 (0.0%) |  | 8 (0.9%) |
| **Language spoken at home^5^** |  |  |  |  |  |  |
| English only | 151 (29.3%) | 203 (39.4%) | 131 (25.4%) | 30 (5.8%) | 0.414 | 515 (59.4%) |
| Language other than English | 102 (29.9%) | 117 (34.3%) | 91 (26.7%) | 31 (9.1%) |  | 341 (39.3%) |
| Don't know/Refused | 4 (36.4%) | 5 (45.5%) | 2 (18.2%) | 0 (0.0%) |  | 11 (1.3%) |
| **Education^5^** |  |  |  |  |  |  |
| High school education or below | 74 (30.8%) | 82 (34.2%) | 64 (26.7%) | 20 (8.3%) | 0.199 | 240 (27.7%) |
| Some college, AA, or vocational/technical degree | 79 (32.4%) | 85 (34.8%) | 67 (27.5%) | 13 (5.3%) |  | 244 (28.1%) |
| Bachelor's, Master's, doctoral, or professional degree | 98 (27.0%) | 151 (41.6%) | 90 (24.8%) | 24 (6.6%) |  | 363 (41.9%) |
| Don't know/Refused | 6 (30.0%) | 7 (35.0%) | 3 (15.0%) | 4 (20.0%) |  | 20 (2.3%) |
| **Employment^5^** |  |  |  |  |  |  |
| Employed | 118 (32.9%) | 147 (41.0%) | 68 (18.9%) | 26 (7.2%) | **0.025** | 359 (41.4%) |
| Unemployed or on disability/leave | 29 (25.4%) | 34 (29.8%) | 39 (34.2%) | 12 (10.5%) |  | 114 (13.1%) |
| Unpaid employment (homemaking, student, volunteer) | 19 (26.0%) | 27 (37.0%) | 25 (34.3%) | 2 (2.7%) |  | 73 (8.4%) |
| Retired | 81 (27.7%) | 106 (36.3%) | 85 (29.1%) | 20 (6.9%) |  | 292 (33.7%) |
| Don't know/Refused | 10 (34.5%) | 11 (37.9%) | 7 (24.1%) | 1 (3.5%) |  | 29 (3.3%) |
| **Income (adjusted for household size)^5^** |  |  |  |  |  |  |
| Less than $25,000 | 74 (30.2%) | 81 (33.1%) | 71 (29.0%) | 19 (7.8%) | 0.432 | 245 (28.3%) |
| $25,000 to $54,999 | 61 (33.3%) | 65 (35.5%) | 47 (25.7%) | 10 (5.5%) |  | 183 (21.1%) |
| $55,000 to $99,999 | 44 (25.0%) | 79 (44.9%) | 43 (24.4%) | 10 (5.7%) |  | 176 (20.3%) |
| $100,000 and above | 28 (36.4%) | 27 (35.1%) | 17 (22.1%) | 5 (6.5%) |  | 77 (8.9%) |
| Don't know/refused | 50 (26.9%) | 73 (39.3%) | 46 (24.7%) | 17 (9.1%) |  | 186 (21.5%) |
| **Home ownership^5^** |  |  |  |  |  |  |
| Owned or being bought by you | 136 (29.1%) | 179 (38.3%) | 120 (25.7%) | 32 (6.9%) | 0.632 | 467 (53.9%) |
| Rented for money | 91 (32.0%) | 95 (33.5%) | 77 (27.1%) | 21 (7.4%) |  | 284 (32.8%) |
| Other/Don't know/Refused | 30 (25.9%) | 51 (44.0%) | 27 (23.3%) | 8 (6.9%) |  | 116 (13.4%) |
| **If you lost all your current income, you could live at your current address and standard of living: ^5^** |  |  |  |  |  |  |
| Less than 1 month | 35 (34.0%) | 28 (27.2%) | 34 (33.0%) | 6 (5.8%) | 0.149 | 103 (11.9%) |
| 1-2 months | 30 (27.8%) | 39 (36.1%) | 29 (26.9%) | 10 (9.3%) |  | 108 (12.5%) |
| 3-6 months | 33 (32.4%) | 42 (41.2%) | 22 (21.6%) | 5 (4.9%) |  | 102 (11.8%) |
| More than 6 months | 93 (27.9%) | 140 (42.0%) | 83 (24.9%) | 17 (5.1%) |  | 333 (38.4%) |
| Don't know/Refused | 66 (29.9%) | 76 (34.4%) | 56 (25.3%) | 23 (10.4%) |  | 221 (25.5%) |
| **I feel confident that I can get medical care without being set back financially: ^5^** |  |  |  |  |  |  |
| Strongly agree | 106 (33.4%) | 126 (39.8%) | 66 (20.8%) | 19 (6.0%) | **0.020** | 317 (36.6%) |
| Agree | 79 (26.3%) | 119 (39.7%) | 82 (27.3%) | 20 (6.7%) |  | 300 (34.6%) |
| Neither agree nor disagree | 23 (20.0%) | 40 (34.8%) | 42 (36.5%) | 10 (8.7%) |  | 115 (13.3%) |
| Disagree | 30 (41.7%) | 21 (29.2%) | 17 (23.6%) | 4 (5.6%) |  | 72 (8.3%) |
| Strongly disagree | 14 (32.6%) | 13 (30.2%) | 12 (27.9%) | 4 (9.3%) |  | 43 (5.0%) |
| Don't know/Refused | 5 (25.0%) | 6 (30.0%) | 5 (25.0%) | 4 (20.0%) |  | 20 (2.3%) |
| **Hospital type^2^** |  |  |  |  |  |  |
| NCI-designated cancer center + ACOS, COC cancer program) | 32 (28.1%) | 45 (39.5%) | 30 (26.3%) | <11 | 0.930 | 114 (13.1%) |
| NCI-designated cancer center only | 19 (32.8%) | 19 (32.3%) | 17 (29.3%) | <11 |  | 58 (6.7%) |
| ACOS, COC cancer program only | 185 (30.1%) | 234 (38.1%) | 152 (24.7%) | 44 (7.2%) |  | 615 (70.9%) |
| Neither NCI nor COC | 21 (26.3%) | 27 (33.8%) | 25 (31.3%) | <11 |  | 80 (9.2%) |

Abbreviations: AJCC, American Joint Committee on Cancer; ACOS, American College of Surgeons; COC, Commission on Cancer; NCI, National Cancer Institute

^1^ Per CCR guidelines, case counts <11 are suppressed for privacy in variables that utilized registry data.

^2^ CCR or CCR-derived variable

^3^ Survey variable, supplemented with available CCR data

^4^ Medically under-resourced patients were defined as being at least one of the following: Non-Hispanic Black, Hispanic, or American/Indian Alaska Native race or ethnicity as identified in CCR; underinsured (no insurance or utilizing any public insurance); living in neighborhoods with the lower 40% of socioeconomic status within California.

^5^ Survey variable

Supplemental Table 2: Distributions of cancer registry or cancer registry-derived sociodemographic variables, compared between those who completed a survey and those who were identified as eligible from the registry.

|  | **Survey Completed** | **Registry Eligible** |  |
| --- | --- | --- | --- |
|  | **N (Col%)^1^** | **N (Col%)^1^** | **p** |
| **Total** | 867 (100%) | 9684 (100%) |  |
| **Age at diagnosis, mean (SD)** | 59.00 (13.78) | 60.86 (13.80) | **<0.001** |
| **Sex** |  |  |  |
| Male | 258 (29.8%) | 1765 (18.2%) | **<0.001** |
| Female | 609 (70.2%) | 7919 (81.8%) |  |
| **Cancer type** |  |  |  |
| Breast cancer | 358 (41.3%) | 6494 (67.1%) | **<0.001** |
| Colorectal cancer | 509 (58.7%) | 3190 (32.9%) |  |
| **AJCC stage** |  |  |  |
| I | 423 (48.8%) | 6131 (63.3%) | **<0.001** |
| II | 209 (24.1%) | 1961 (20.2%) |  |
| III | 235 (27.1%) | 1592 (16.4%) |  |
| **Year of diagnosis** |  |  |  |
| 2019 | 396 (45.7%) | 5425 (56.0%) | **<0.001** |
| 2020 | 378 (43.6%) | 3560 (36.8%) |  |
| 2021 | 93 (10.7%) | 699 (7.2%) |  |
| **Medically under-resourced^2^** |  |  |  |
| No | 302 (34.8%) | 6867 (65.1%) | **<0.001** |
| Yes | 565 (65.2%) | 3684 (34.9%) |  |
| **Race/ethnicity group** |  |  |  |
| American Indian/Alaska Native | <11 | 40 (0.4%) | **<0.001** |
| Asian American/Pacific Islander | 166 (19.1%) | 2668 (27.6%) |  |
| Hispanic | 250 (28.8%) | 1449 (15.0%) |  |
| NH Black | 112 (12.9%) | 592 (6.1%) |  |
| NH White | 329 (37.9%) | 4890 (50.5%) |  |
| Unknown | <11 | 45 (0.5%) |  |
| **Payer status** |  |  |  |
| No insurance | 10 (1.2%) | 42 (0.4%) | **<0.001** |
| Private only | 450 (51.9%) | 5506 (56.9%) |  |
| Medicare only or Medicare + private | 204 (23.5%) | 2841 (29.3%) |  |
| Any public/Medicaid/military | 192 (22.1%) | 1181 (12.2%) |  |
| Unknown | 11 (1.3%) | 114 (1.2%) |  |
| **Neighborhood SES (census tract)** |  |  |  |
| Q1 (lowest nSES) | 44 (5.1%) | 297 (2.8%) | **<0.001** |
| Q2 | 96 (11.1%) | 737 (7.0%) |  |
| Q3 | 131 (15.1%) | 1354 (12.8%) |  |
| Q4 | 205 (23.6%) | 2460 (23.3%) |  |
| Q5 (highest nSES) | 348 (40.1%) | 5181 (49.1%) |  |
| Unknown/Missing | 43 (5.0%) | 522 (4.9%) |  |
| **Marital status** |  |  |  |
| Single, never married | 188 (21.7%) | 1761 (18.2%) | 0.008 |
| Married | 504 (58.1%) | 5840 (60.3%) |  |
| Separated | <11 | 96 (1.0%) |  |
| Divorced | 78 (9.0%) | 686 (7.1%) |  |
| Widowed | 59 (6.8%) | 786 (8.1%) |  |
| Unmarried or domestic partner | <11 | 85 (0.9%) |  |
| Unknown | 29 (3.3%) | 430 (4.4%) |  |
| **Marital status (simplified)** |  |  |  |
| Partnered | 507 (58.5%) | 5925 (61.2%) | 0.039 |
| Unpartnered | 331 (38.2%) | 3329 (34.4%) |  |
| Unknown | 29 (3.3%) | 430 (4.4%) |  |
| **Hospital type** |  |  |  |
| NCI-designated cancer center + ACOS, COC cancer program | 114 (13.1%) | 1106 (11.4%) | 0.438 |
| NCI-designated cancer center only | 58 (6.7%) | 712 (7.4%) |  |
| ACOS, COC cancer program only | 615 (70.9%) | 6934 (71.6%) |  |
| Neither NCI nor COC | 80 (9.2%) | 932 (9.6%) |  |

Abbreviations: AJCC, American Joint Committee on Cancer; ACOS, American College of Surgeons; COC, Commission on Cancer; NCI, National Cancer Institute

Bolded associations were significant at a Bonferroni corrected alpha=0.004

^1^ Per CCR guidelines, case counts <11 are suppressed for privacy.

^2^ Medically under-resourced patients were defined as being at least one of the following: Non-Hispanic Black, Hispanic, or American/Indian Alaska Native race or ethnicity; underinsured (no insurance or utilizing any public insurance); living in neighborhoods with the lower 40% of socioeconomic status within California.

Supplemental Table 3: Distributions of cancer registry or cancer registry-derived sociodemographic variables, compared between those who completed a survey and those who were identified as eligible from the registry, among medically under-resourced participants only.

|  | **Survey Completed** | **Registry Eligible** |  |
| --- | --- | --- | --- |
|  | **N (Col%)^1^** | **N (Col%)^1^** | **p** |
| **Total** | 565 (100%) | 3119 (100%) |  |
| **Age at diagnosis, mean (SD)** | 58.15 (13.51) | 59.83 (13.67) | 0.007 |
| **Sex** |  |  |  |
| Male | 148 (26.2%) | 654 (21.0%) | 0.006 |
| Female | 417 (73.8%) | 2465 (79.0%) |  |
| **Cancer type** |  |  |  |
| Breast cancer | 252 (44.6%) | 1912 (61.3%) | **<0.001** |
| Colorectal cancer | 313 (55.4%) | 1207 (38.7%) |  |
| **AJCC stage** |  |  |  |
| I | 273 (48.3%) | 1726 (55.3%) | **0.003** |
| II | 141 (25.0%) | 738 (23.7%) |  |
| III | 151 (26.7%) | 655 (21.0%) |  |
| **Year of diagnosis** |  |  |  |
| 2019 | 265 (46.9%) | 1730 (55.5%) | **<0.001** |
| 2020 | 235 (41.6%) | 1130 (36.2%) |  |
| 2021 | 65 (11.5%) | 259 (8.3%) |  |
| **Race/ethnicity group** |  |  |  |
| American Indian/Alaska Native | 8 (1.4%) | 34 (1.1%) | 0.459 |
| Asian American/Pacific Islander | 93 (16.5%) | 562 (18.0%) |  |
| Hispanic | 249 (44.1%) | 1438 (46.1%) |  |
| NH Black | 112 (19.8%) | 587 (18.8%) |  |
| NH White | 103 (18.2%) | 492 (15.8%) |  |
| Unknown/Missing | 0 (0.0%) | <11 |  |
| **Payer status** |  |  |  |
| No insurance | <11 | 39 (1.3%) | 0.436 |
| Private only | 254 (45.0%) | 1313 (42.1%) |  |
| Medicare only or Medicare + private | 108 (19.1%) | 598 (19.2%) |  |
| Any public/Medicaid/military | 188 (33.3%) | 1127 (36.1%) |  |
| Unknown | <11 | 42 (1.3%) |  |
| **Neighborhood SES (census tract)** |  |  |  |
| Q1 (lowest nSES) | 42 (7.4%) | 242 (7.8%) | 0.391 |
| Q2 | 93 (16.5%) | 588 (18.9%) |  |
| Q3 | 107 (18.9%) | 630 (20.2%) |  |
| Q4 | 148 (26.2%) | 706 (22.6%) |  |
| Q5 (highest nSES) | 149 (26.4%) | 788 (25.3%) |  |
| Unknown/Missing | 26 (4.6%) | 165 (5.3%) |  |
| **Marital status** |  |  |  |
| Single, never married | 131 (23.2%) | 766 (24.6%) | 0.005 |
| Married | 314 (55.6%) | 1566 (50.2%) |  |
| Separated | 4 (0.7%) | 57 (1.8%) |  |
| Divorced | 58 (10.3%) | 250 (8.0%) |  |
| Widowed | 34 (6.0%) | 290 (9.3%) |  |
| Unmarried or domestic partner | 2 (0.4%) | 24 (0.8%) |  |
| Unknown | 22 (3.9%) | 166 (5.3%) |  |
| **Marital status (simplified)** |  |  |  |
| Partnered | 227 (40.2%) | 1363 (43.7%) | 0.062 |
| Unpartnered | 316 (55.9%) | 1590 (51.0%) |  |
| Unknown | 22 (3.9%) | 166 (5.3%) |  |
| **Hospital type** |  |  |  |
| NCI-designated cancer center + ACOS, COC cancer program | 394 (69.7%) | 2197 (70.4%) | 0.195 |
| NCI-designated cancer center only | 79 (14.0%) | 351 (11.3%) |  |
| ACOS, COC cancer program only | 33 (5.8%) | 182 (5.8%) |  |
| Neither NCI nor COC | 59 (10.4%) | 389 (12.5%) |  |

Abbreviations: AJCC, American Joint Committee on Cancer; ACOS, American College of Surgeons; COC, Commission on Cancer; NCI, National Cancer Institute

Bolded associations were significant at a Bonferroni corrected alpha=0.005

^1^Per CCR guidelines, case counts <11 are suppressed for privacy.

Supplemental Table 4: Distributions of CCR or CCR-derived sociodemographic variables, compared between those who completed a survey and those who were identified for outreach but did not complete a survey due to refusal, ineligibility, or loss to follow up.

|  | **Survey Completed** | **Non-Responder** |  |
| --- | --- | --- | --- |
|  | **N (Col%)^1^** | **N (Col%)^1^** | **p** |
| **Total** | 867 (100%) | 1812 (100%) |  |
| **Age at diagnosis, mean (SD)** | 59.00 (13.78) | 63.33 (13.71) | **<0.001** |
| **Sex** |  |  |  |
| Male | 258 (29.8%) | 691 (38.1%) | **<0.001** |
| Female | 609 (70.2%) | 1121 (61.9%) |  |
| **Cancer type** |  |  |  |
| Breast cancer | 358 (41.3%) | 568 (31.3%) | **<0.001** |
| Colorectal cancer | 509 (58.7%) | 1244 (68.7%) |  |
| **AJCC stage** |  |  |  |
| I | 423 (48.8%) | 814 (44.9%) | 0.110 |
| II | 209 (24.1%) | 497 (27.4%) |  |
| III | 235 (27.1%) | 501 (27.6%) |  |
| **Year of diagnosis** |  |  |  |
| 2019 | 396 (45.7%) | 898 (49.6%) | 0.005 |
| 2020 | 378 (43.6%) | 676 (37.3%) |  |
| 2021 | 93 (10.7%) | 238 (13.1%) |  |
| **Medically under-resourced^2^** |  |  |  |
| No | 302 (34.8%) | 502 (27.7%) | **<0.001** |
| Yes | 565 (65.2%) | 1310 (72.3%) |  |
| **Race/ethnicity group** |  |  |  |
| American Indian/Alaska Native | <11 | 11 (0.6%) | **<0.001** |
| Asian American/Pacific Islander | 166 (19.1%) | 443 (24.4%) |  |
| Hispanic | 250 (28.8%) | 597 (32.9%) |  |
| NH Black | 112 (12.9%) | 225 (12.4%) |  |
| NH White | 329 (37.9%) | 526 (29.0%) |  |
| Unknown/Missing | <11 | <11 |  |
| **Payer status** |  |  |  |
| No insurance | <11 | 18 (1.0%) | **<0.001** |
| Private only | 450 (51.9%) | 782 (43.2%) |  |
| Medicare only or Medicare + private | 204 (23.5%) | 480 (26.5%) |  |
| Any public/Medicaid/military | 192 (22.1%) | 505 (27.9%) |  |
| Unknown | 11 (1.3%) | 27 (1.5%) |  |
| **Neighborhood SES (census tract)** |  |  |  |
| Q1 (lowest nSES) | 44 (5.1%) | 96 (5.3%) | 0.011 |
| Q2 | 96 (11.1%) | 268 (14.8%) |  |
| Q3 | 131 (15.1%) | 313 (17.3%) |  |
| Q4 | 205 (23.6%) | 415 (22.9%) |  |
| Q5 (highest nSES) | 348 (40.1%) | 615 (33.9%) |  |
| Unknown/Missing | 43 (5.0%) | 105 (5.8%) |  |
| **Marital status** |  |  |  |
| Single, never married | 188 (21.7%) | 397 (21.9%) | **<0.001** |
| Married | 504 (58.1%) | 960 (53.0%) |  |
| Separated | <11 | 31 (1.7%) |  |
| Divorced | 78 (9.0%) | 122 (6.7%) |  |
| Widowed | 59 (6.8%) | 192 (10.6%) |  |
| Unmarried or domestic partner | <11 | 12 (0.7%) |  |
| Unknown | 29 (3.3%) | 98 (5.4%) |  |
| **Marital status (simplified)** |  |  |  |
| Partnered | 507 (58.5%) | 972 (53.6) | 0.012 |
| Unpartnered | 331 (38.2%) | 742 (40.9%) |  |
| Unknown | 29 (3.3%) | 98 (5.4%) |  |
| **Hospital type** |  |  |  |
| NCI-designated cancer center + ACOS, COC cancer program | 114 (13.1%) | 178 (9.8%) | **<0.001** |
| NCI-designated cancer center only | 58 (6.7%) | 117 (6.5%) |  |
| ACOS, COC cancer program only | 615 (70.9%) | 1265 (69.8%) |  |
| Neither NCI nor COC | 80 (9.2%) | 252 (13.9%) |  |

Abbreviations: AJCC, American Joint Committee on Cancer; ACOS, American College of Surgeons; COC, Commission on Cancer; NCI, National Cancer Institute

Bolded associations were significant at a Bonferroni corrected alpha=0.004

^1^Per CCR guidelines, case counts <11 are suppressed for privacy.

^2^Medically under-resourced patients were defined as being at least one of the following: Non-Hispanic Black, Hispanic, or American/Indian Alaska Native race or ethnicity; underinsured (no insurance or utilizing any public insurance); living in neighborhoods with the lower 40% of socioeconomic status within California.
